# Efficacy and safety of remimazolam for procedural sedation during ultrasound-guided transversus abdominis plane block and rectus sheath block in patients undergoing abdominal tumor surgery: a single-center randomized controlled trial

**DOI:** 10.1186/s12871-022-01927-8

**Published:** 2022-12-08

**Authors:** Yimin Xiao, Ran Wei, Lanren Chen, Yunfei Chen, Lingsuo Kong

**Affiliations:** Department of Anesthesiology, Anhui Provincial Cancer Hospital, Huanhu East road 107, Shushan District, 230022 Hefei, China

**Keywords:** Remimazolam, Procedural sedation, Ultrasound-guided nerve block, Abdominal tumor surgery, Clinical anesthesia

## Abstract

**Background:**

To explore the efficacy and safety of remimazolam for procedural sedation during ultrasound-guided nerve block administration in patients undergoing abdominal tumor surgery, in order to improve and optimize remimazolam use in procedural sedation and clinical anesthesia.

**Methods:**

The enrolled patients were randomly divided into three groups: 50 patients in the remimazolam group (R group), 50 patients in the dexmedetomidine group (D group), and 50 patients in the midazolam group (M group). Before administering an ultrasound-guided nerve block, all patients received sufentanil AND remimazolam or midazolam or dexmedetomidine. Remimazolam 5 mg was administered intravenously in group R, dexmedetomidine 0.6 µg/kg was administered intravenously in group D, and midazolam 0.025 mg/kg was administered intravenously in group M. Sedation was evaluated by the Modified Observer’s Assessment of Alertness and Sedation scale.When the Modified Observer’s Alertness/Sedation (MOAA/S) score was ≤ 2, block operation was started. If the target sedation level was not reached, rescue sedatives of remimazolam 2.5 mg may be intravenously given in group R, dexmedetomidine 0.4 µg/kg be intravenously given in group D, 0.01 mg/kg midazolam may be intravenously given in Group M. Hemodynamic indicators (systolic and diastolic blood pressure, heart rate), pulse oxygen saturation, depth of anesthesia (Narcotrend), MOAA/S,and the incidences of hypoxemia, injection pain, bradycardia and requirement for rescue sedatives were monitored and recorded.

**Results:**

Compared with the control groups (midazolam and dexmedetomidine groups), the Narcotrend index and MOAA/S decreased more in the remimazolam group (*P* < 0.01). Compared with the control groups, the incidence of hypoxemia and injection pain was slightly higher in the remimazolam group, but the difference was not statistically significant (*P* > 0.05). Compared with the dexmedetomidine group, the incidence of bradycardia was significantly lower in the remimazolam group.

**Conclusion:**

Remimazolam can be used safely for procedural sedation during ultrasound-guided nerve block administration in patients undergoing abdominal tumor surgery. The sedation effect is better than that with either midazolam or dexmedetomidine, and sedation can be achieved quickly without obvious hemodynamic fluctuations. Remimazolam is associated with better heart rate stability, and slightly higher incidences of hypoxemia and injection pain than are midazolam and dexmedetomidine (no statistically significant difference). The higher incidence of hypoxemia with remimazolam may be related to enhanced sufentanil opioid analgesia, and the mechanism of injection pain with remimazolam must be studied further and clarified.

**Trial registration:**

This study was approved by the Ethics Committee of Anhui Provincial Cancer Hospital (Ethical Review 2021, No. 23) and registered at https://www.chictr.org.cn (ChiCTR2000035388). The pre-registration time of this experiment is 09/08/2020, due to ethical committee of the hospital met irregularly,the ethical approval time is 21/06/2021. The recruitment of patients began after the ethical approval (21/06/2021) and registration update (06/07/2021).The study protocol followed the CONSORT guidelines. The study protocol was performed in the relevant guidelines.

## Background

Benzodiazepine sedative-hypnotics are commonly used intravenous anesthetics [1]. Remimazolam is a new type of benzodiazepine with fast onset, short maintenance and recovery time, no accumulation, metabolism independent of liver and kidney function, no serious side effects, and good clinical application prospects [2]. The existing literature suggests that the clinical application of remimazolam is divided into four categories: preoperative medication; compounded with opioids for anesthesia in some procedural endoscopy situations, to exert a sedative effect [3]; in total intravenous anesthesia for induction and maintenance – a small number of studies have shown that remimazolam can be used for induction and maintenance of general anesthesia, with higher safety than with propofol, lower incidence of hypotension, fewer vasopressor doses, and lower incidence of injection pain; and sedation in intensive care patients [4].

The current number of clinical studies on remimazolam is relatively limited, and most focused on the sedative effect of remimazolam compounded with opioids in outpatient procedural endoscopy (such as colonoscopy, bronchoscopy, and gastrointestinal endoscopy). However, remimazolam is also used in clinical anesthesia (e.g., for procedural sedation during ultrasound-guided nerve block administration in patients undergoing abdominal tumor surgery). The use of remimazolam requires additional scientific experimental results and data support. Additionally, overall, the application of clinical anesthesia requires innovation and exploration to maximize the benefits for patients’ comfortable medical care.

This study aimed to explore the efficacy and safety of remimazolam for procedural sedation during ultrasound-guided nerve block administration in patients undergoing abdominal tumor surgery, in order to improve and optimize remimazolam use in clinical anesthesia.

## Methods

### Ethics and registration

This study was approved by the Ethics Committee of Anhui Provincial Cancer Hospital (Ethical Review 2021, No. 23) and registered at https://www.chictr.org.cn (ChiCTR2000035388). The pre-registration time of this experiment is 09/08/2020, due to ethical committee of the hospital met irregularly,the ethical approval time is 21/06/2021. The recruitment of patients began after the ethical approval (21/06/2021) and registration update (06/07/2021).The study protocol followed the CONSORT guidelines. The study protocol was performed in the relevant guidelines. The study met the provisions of the Declaration of Helsinki.

This randomized, controlled, double-blind study enrolled patients scheduled for abdominal tumor surgery at Anhui Provincial Cancer Hospital (Hefei, China); all patients provided written informed consent.

### Patient inclusion and exclusion criteria

All patients were aged 18–75 years, had an American Society of Anesthesiologists physical status of I–III, and were scheduled for abdominal tumor surgery. The exclusion criteria were as follows: declined ultrasound-guided nerve block, presence of obvious organ dysfunction, severe electrolyte imbalance, infection at the puncture site, abnormal blood coagulation profile before surgery, receiving an antiplatelet agent, hypersensitivity to local anesthetics, or hypersensitivity or allergy to the drugs in this study. Patients were also excluded if they had central neuropathy, body mass index > 35 kg/m^2^, or a history of abuse of benzodiazepines and/or opioids.

### Randomization

After obtaining written informed consent, all patients were randomized to one of three groups (remimazolam, midazolam, or dexmedetomidine) using computer-generated random numbers and a 1:1:1 allocation ratio. Allocation concealment was fulfilled by an assistant not involved in the study, and randomization was achieved using sequentially numbered, sealed, opaque envelopes. One envelope was opened after each patient’s arrival to the operation room.

### Technique

All patients fasted routinely before surgery. Patients were placed in a standard supine position to administer an ultrasound-guided nerve block (transversus abdominis plane block (TAPB) or rectus sheath block (RSB)) followed by standardized monitoring,including electrocardiogram (ECG),noninvasive blood pressure (NIBP) including systolic blood pressure (SBP) and diastolic blood pressure (DBP),pulse oxygen saturation(SpO_2_), heart rate (HR) and Narcotrend,MOAA/S.

**Fig. 1:**
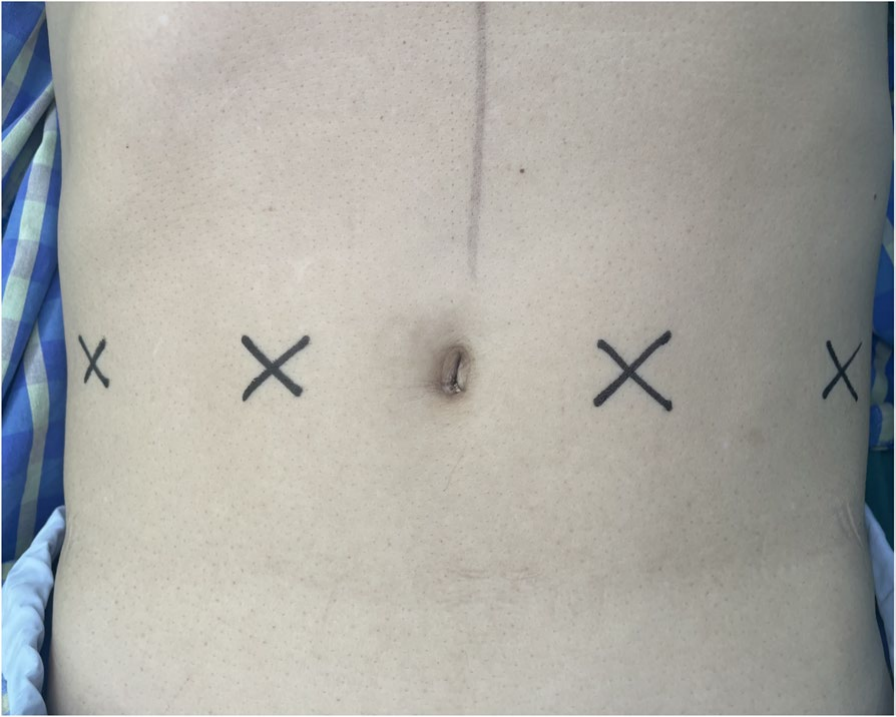
Injection sites on the left and right in the abdominal wall for ultra-sound guided block

TAPB and RSB were performed by the same two anesthesiologists, who had considerable experience of more than 5 years performing ultrasound-guided nerve blocks. Real-time ultrasonography (Mindray Ultrasound System; Mindray Medical International, Shenzhen, China) was used when performing the blocks. The nerve block procedure was divided into 4 injection sites on the left and right in the abdominal wall to ensure the diffusion of the drug (Fig. 1).

**Fig. 2:**
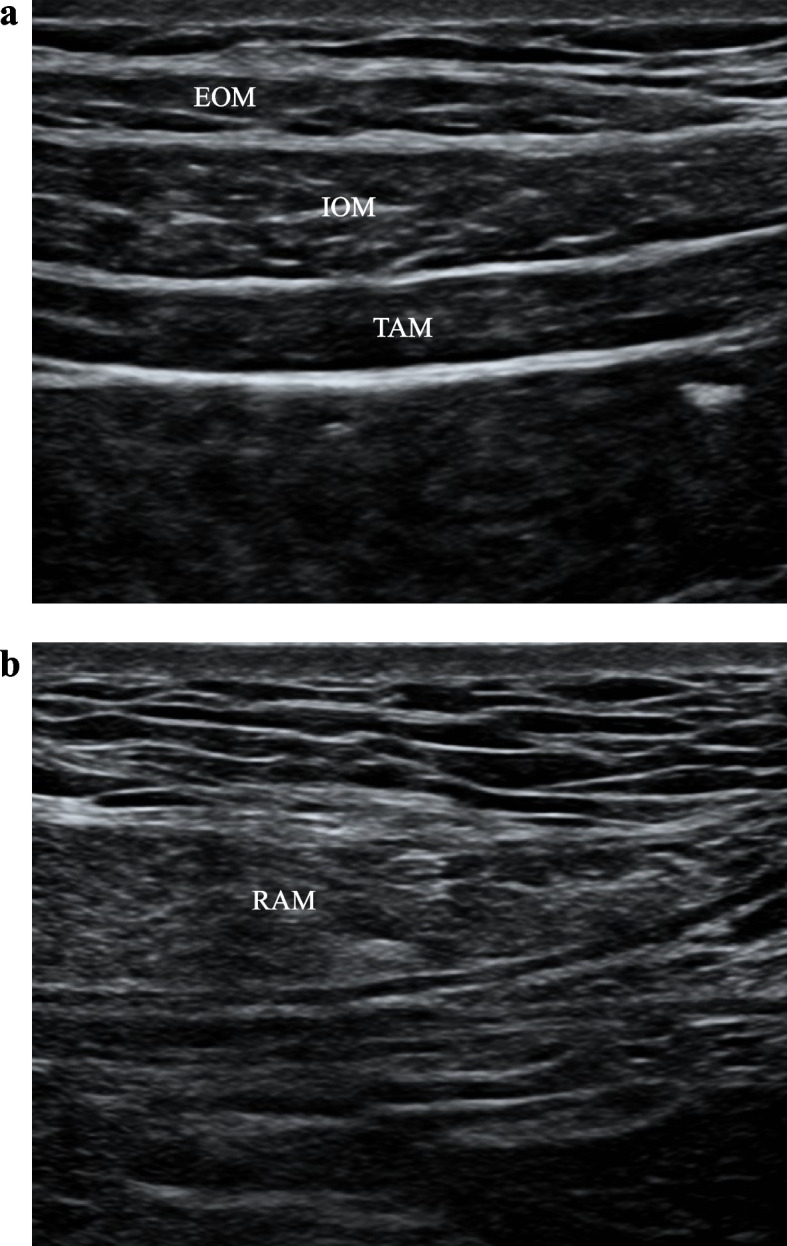
**a** Ultrasonographic image taken before the transversus abdominis plane block (TAPB). **b** Ultrasonographic image taken before the rectus sheath block (RSB)

For TAPB (Fig. 2a), a high-frequency linear ultrasound probe was placed transversely on the midaxillary line between the iliac crest and the costal margin [5]. Then, the needle (Stimuplex D; B. Braun Melsungen AG, Melsungen, Germany) was inserted when the TAP was identified. When the tip of the needle was in the TAP, 2 mL of normal saline was injected to adjust and ensure the needle’s position. Next, 20 mL of 0.5% ropivacaine was administered bilaterally (Fig. 2b).

**Fig. 3:**
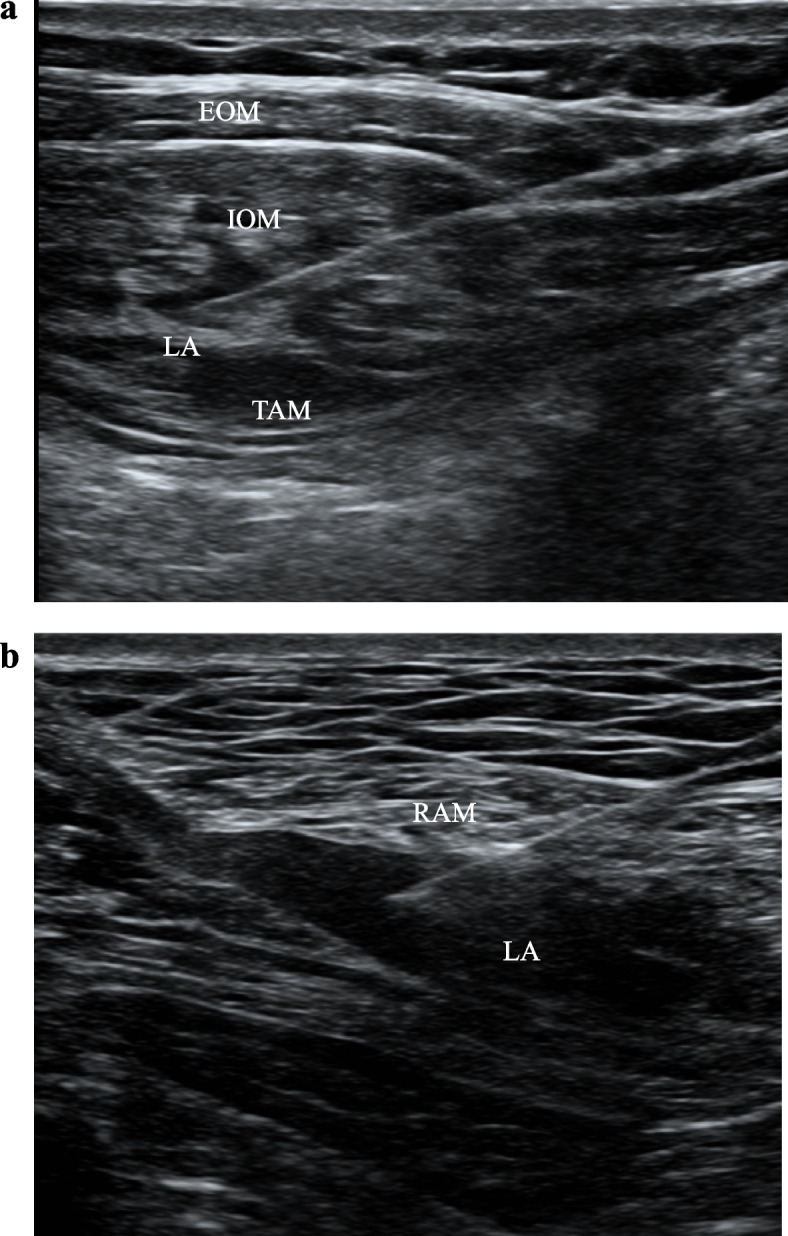
**a **Ultrasonographic image taken after the transversus abdominis plane block (TAPB). **b** Ultrasonographic image taken after the rectus sheath block (RSB). EOM – external oblique muscle; IOM – internal oblique muscle; TAM – transverse abdominal muscle; LA – local anesthetics; RAM – rectus abdominis muscle

RSB (Fig. 3a) was performed on both sides of the linea alba under ultrasound guidance [6]. The probe was placed transversely on the rectus abdominis, and the needle was inserted using ultrasound guidance until the tip was in the plane between the rectus abdominis and the posterior sheath of the rectus abdominis [7]. All patients undergoing RSB received 40 mL of 0.5% ropivacaine (Fig. 3b).

Twenty min after finishing these blocks, pinprick tests were performed bilaterally on the area from T6 to T12, includes the area 3 cm lateral from the midline incision to evaluate the anterior cutaneous branch innervation area, and the mid-axillary line to evaluate the lateral cutaneous branch innervation area. 0 = loss of pinprick sensation, 1 = decreased pinprick sensation, 2 = normal pinprick sensation. An effective sensory block was defined as a score of 0 or 1. Patients were excluded from the study if sensory blockade was unsuccessful.

### Grouping and intervention

An assistant who was not involved in the study and did not participate perioperatively prepared the study drugs. Before administering the ultrasound-guided nerve block, all patients were given sufentanil (Sufentanil Citrate Injection, Yichang Humanwell Pharmaceutical Co., LTD., China) 5 µg. Additionally, remimazolam (Remimazolam Tosilate for Injection, Yichang Humanwell Pharmaceutical Co., LTD., China) mg was administered intravenously in group R, dexmedetomidine (Dexmedetomidine Hydrochloride Injection, Yangtze River Pharmaceutical Group, China) 0.6 µg/kg was administered intravenously in group D, and midazolam (Midazolam injection; Enhua Pharmaceutical, China) 0.025 mg/kg was administered intravenously in group M. Each patient’s sedation level was evaluated using the Modified Observer’s Assessment of Alertness and Sedation scale. If the target sedation level was not reached, rescue sedatives of remimazolam 2.5 mg may be intravenously given in group R, dexmedetomidine 0.4 µg/kg be intravenously given in group D, 0.01 mg/kg midazolam may be intravenously given in Group M.

Patient hemodynamic indicators, namely systolic and diastolic blood pressure; heart rate (HR); mean arterial pressure (MAP), which was derived from the following equation: MAP = (systolic blood pressure + 2 × diastolic blood pressure)/3; pulse oxygen saturation (SpO_2_); Narcotrend (depth of anesthesia); MOAA/S, and the incidences of hypoxemia, injection pain, bradycardia and requirement for rescue sedatives were monitored and recorded.

### Outcomes

#### Primary outcomes

The primary outcomes of this study were the mean arterial pressure (MAP), heart rate (HR), pulse oxygen saturation (SpO_2_), MOAA/S score and Narcotrend value of the three groups.

#### Secondary outcomes

The secondary outcome of this study the incidences of hypoxemia, injection pain, bradycardia and requirement for rescue sedatives in the three groups during the nerve block.

### Sample size and statistical analysis

Calculations of the sample size were performed using an online power sample size calculator based on our previous pilot study showing decreased Narcotrend index values for patients under sedation with dexmedetomidine and with midazolam (40.5 ± 7.0 and 44.3 ± 7.8, respectively) compared with patients under sedation with remimazolam (55.5 ± 7.3). The sample size was calculated as 18 per group at a power of 80% and a two-tailed α-error of 5%. According to the clinical experience related to this experiment, subjects may withdraw from the study due to changes in surgery or anesthesia protocol, abnormal parameter collection due to poor device contact, poor subject compliance, etc.We enrolled 150 patients in total (N = 50/group) to account for potential study dropouts to ensure the final effective sample size.

Statistical analysis was performed using SPSS Statistics 17.0.1 (SPSS Inc., Chicago, IL). Normality test in SPSS statistics software was used for data analysis to determine whether the data were in accordance with a normal distribution. Normally distributed continuous variables are presented as the mean ± standard deviation and were analysed using Student’s t test. The MannWhitney U test was used for non-normally distributed continuous variables. Hemodynamic parameters were compared by repeated measures ANOVA. Categorical variables are expressed as a frequency (percentage) and were analysed using the Pearson chi-square test. The Wilcoxon Signed-Rank test was used to compare continuous variables. A P value < 0.05 was considered to indicate statistical significance.

## Results

**Fig. 4:**
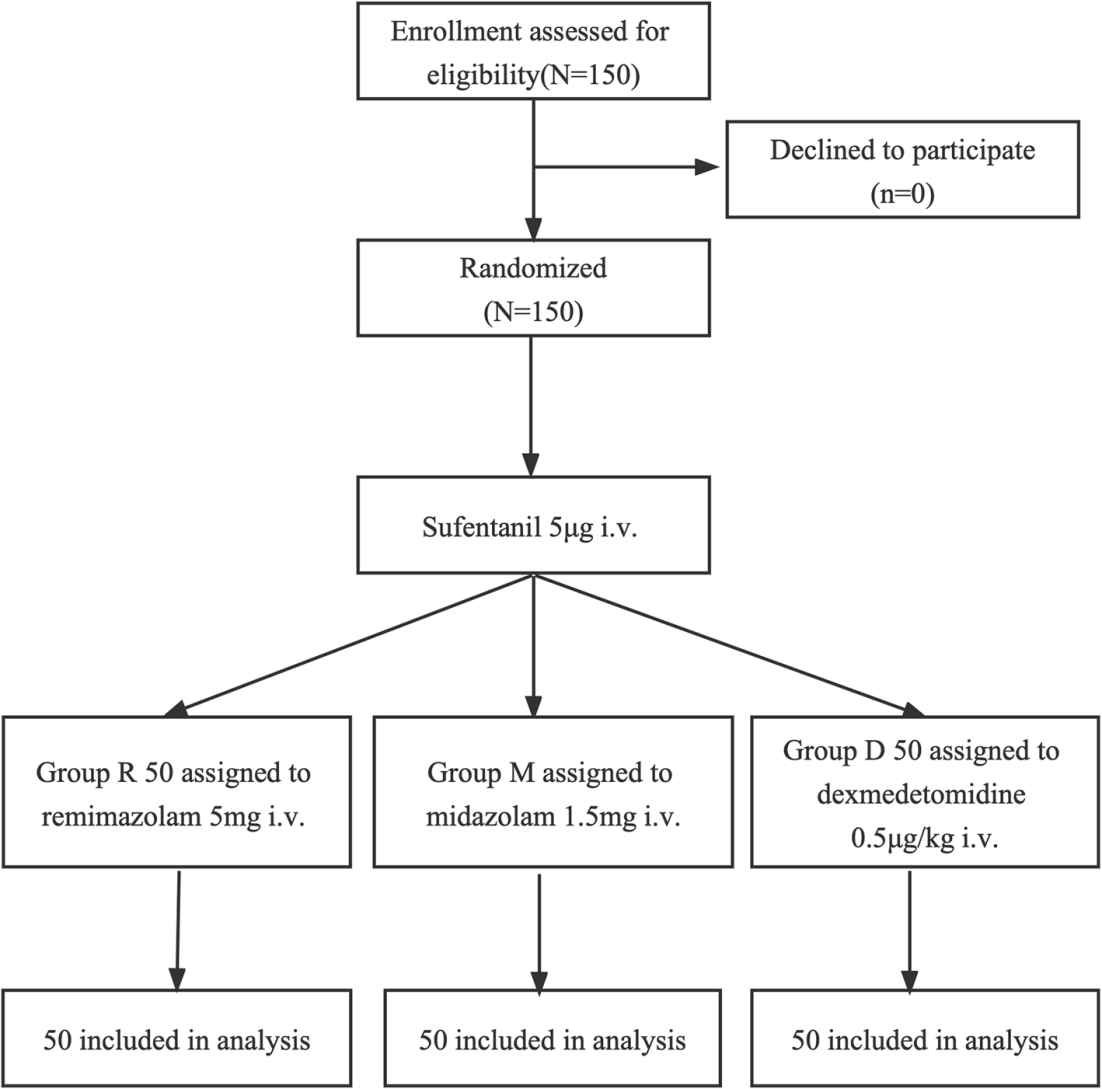
Study flowchart. R, remimazolam; M, midazolam; D, dexmedetomidine

The study flowchart is depicted in Fig. 4.

Table 1 contains the patients’ data. There were no significant differences in the patient characteristics data

**Table 1:** Patient characteristics data

Variables	Group R	Group M	Group D	*P* value
Sex				0.602
Male	24(48.0)	27(54.0)	22(44.0)	
Female	26(52.0)	23(46.0)	28(56.0)	
Age (years)	56.1(7.8)	58.0(10.4)	57.9(9.5)	0.503
Height (cm)	162.1(5.5)	162.4(4.5)	161.7(5.0)	0.825
Weight (kg)	58.8(5.7)	57.6(6.2)	57.8(5.7)	0.550
BMI(kg/m^2^)	22.4(2.3)	21.9(2.6)	22.1(2.5)	0.593
Duration of the block(min)	9.3(2.5)	9.1(2.0)	9.5(2.3)	0.768

Data represent mean (standard deviation) or number (%). *P*<0.05 was considered a statistically significant difference. *R* Remimazolam, *M* Midazolam, *D* Dexmedetomidine, *BMI* Body mass index

Table 2 shows the changes in the patients’ vital signs in the three groups during the nerve block. There were significant differences in MAP, HR, SpO_2_, and the Narcotrend index,MOAA/S at different time points (F = 121.1, 25.286, 540.8, and 221.1, 321.7 ,respectively; all, *P* < 0.001). There were also significant differences in MAP, HR, SpO_2_, and the Narcotrend index, MOAA/S among the groups (F = 7.632, 3.579, 6.81, and 142.35, 28.67 ,respectively; all, *P* < 0.05). The changes in the trends for MAP and HR in group D were significantly different from those in group R and group M (both, *P* < 0.05). The changes in the trends for SpO_2_ and the Narcotrend index and MOAA/S differed significantly between the groups (all, *P* < 0.05). Compared with midazolam and dexmedetomidine, remimazolam administration achieved the desired depth of sedation quickly and was associated with rapid sedation onset and recovery and stable hemodynamics without increasing the incidence of respiratory depression.

**Table 2:** Vital signs of the patients during the nerve blocks

Variables	Time point	Group R	Group M	Group D	F value	*P* value
MAP	Baseline	77.5(3.8)	77.7(3.3)	77.9(3.2)	0.109	0.896
	1min	73.1(3.5)	73.5(3.6)	72.4(3.6)	1.091	0.339
	3min	72.1(3.7)	73.1(3.9)	75.4(4.7)	8.224	0.000
	5min	74.2(3.8)	73.5(4.5)	77.8(4.4)	14.694	0.000
	10min	75.7(3.7)	74.4(3.6)	78.5(4.2)	14.593	0.000
	15min	77.3(3.3)	76.5(2.8)	77.5(4.4)	1.087	0.340
HR	Baseline	69.6(3.6)	68.6(3.8)	68.7(3.6)	1.079	0.342
	1min	68.2(3.7)	69.3(4.0)	66.8(5.4)	3.865	0.023
	3min	67.2(3.5)	65.5(4.5)	63.6(4.9)	8.236	0.000
	5min	68.3(3.5)	67.5(5.5)	65.5(4.9)	4.667	0.011
	10min	67.9(3.9)	70.5(5.0)	68.7(5.9)	3.582	0.030
	15min	70.2(2.9)	71.1(3.6)	69.1(4.6)	3.561	0.031
SpO_2_	Baseline	97.2(0.9)	97(0.9)	97(1.0)	0.502	0.607
	1min	93.1(1.7)	94.0(1.5)	94.1(1.3)	6.346	0.002
	3min	92.6(2.3)	92.9(1.5)	92.3(1.5)	1.153	0.319
	5min	93.3(2.1)	92.2(2.0)	91.1(1.7)	15.822	0.000
	10min	94.7(1.9)	92.8(2.1)	92.0(1.8)	24.815	0.000
	15min	95.7(1.6)	94.3(1.6)	94.0(1.3)	17.547	0.000
Narcotrend	Baseline	98.1(0.5)	97.9(0.5)	98.0(0.4)	0.894	0.411
	1min	59.9(1.1)	84.6(1.4)	90.1(0.7)	10190	0.000
	3min	80.9(0.6)	78.6(1.4)	79.0(1.0)	38.141	0.000
	5min	88.1(1.0)	84.4(6.0)	74.1(1.1)	199.947	0.000
	10min	88.3(0.9)	75.1(1.0)	69.9(1.1)	4217	0.000
	15min	89.9(1.1)	73.0(0.98)	70.0(1.0)	5369	0.000
MOAA/S	Baseline	4.56(0.5)	4.64(0.5)	4.16(0.5)	9.483	0.000
	1min	1.10(0.5)	2.04(0.2)	2.86(0.4)	171.775	0.000
	3min	1.90(0.3)	1.86(0.5)	1.96(0.5)	121.381	0.000
	5min	1.96(0.5)	2.74(0.5)	2.46(0.4)	97.666	0.000
	10min	3.24(0.4)	2.94(0.5)	2.56(0.2)	351.997	0.000
	15min	4.58(0.5)	4.01(0.5)	4.07(0.5)	13.617	0.000

*MAP* Mean arterial pressure, *HR* Heart rate, *SpO*_*2*_ Pulse oxygen saturation, *R* Remimazolam, *M* Midazolam, *D* Dexmedetomidine. MOAA/S,Modified Observer’s Alertness/Sedation

Table 3 shows the incidences of hypoxemia, injection pain, bradycardia and requirement for rescue sedatives in the three groups during the nerve block. These events were treated by injecting ephedrine or atropine intravenously, or mask ventilating with oxygen. There was no significant difference in the incidence of hypoxemia or injection pain or requirement for rescue sedatives between the groups. However, the incidence of bradycardia in group D was significantly higher than that in group R and group M (*P* < 0.05). No patient experienced block failure, subjective symptoms of local anesthetic toxicity, infection, or hematoma at needle insertion site.

**Table 3:** Incidences of hypoxemia, injection pain, bradycardia and requirement for rescue sedatives during the nerve block

Variables	Group R	Group M	Group D	*P* value
Hypoxemia	15(30.0)	12(24.0)	10(20.0)	0.542
Injection pain	12(24.0)	9(18.0)	7(14.0)	0.473
Bradycardia	8(16.0)	10(20.0)	20(40.0)^*#^	0.015
Requirement for rescue sedatives	4(8.0)	6(12)	5(10)	0.199

Injection pain was evaluated subjectively with patients verbally reporting their pain level after the first injection.

Hypoxemia = SpO_2_ <90%; Bradycardia = HR <60 bpm.

Data are presented as number (%). *P*<0.05 was considered a statistically significant difference. *, *P*<0.05 compared with group R; #, *P*<0.05 compared with group M.

*R* Remimazolam, *M* Midazolam, *D* Dexmedetomidine, *SpO*_*2*_ Pulse oxygen saturation, *HR* heart rate.

## Discussion

Remimazolam, a new ultrashort-acting y-aminobutyric acid A (GABA_A_) receptor agonist, was approved for the induction and maintenance of general anesthesia in adults on 23 January 2020 in Japan [8, 9]. Remimazolam was approved by the US Food and Drug Administration on 3 July 2020 for injection to achieve induction and maintenance of procedural sedation in adults undergoing procedures lasting 30 min or less and by the Chinese National Medical Products Administration on 20 July 2020 for use in procedural sedation [10]. Procedural sedation comprises the administration of hypnotic agents or techniques to enable the effective completion of a diagnostic or therapeutic procedure, which may be otherwise painful or uncomfortable for patients [4, 11].

Clinical procedures often result in patient anxiety, fear, and physical or emotional distress owing to the possibility of pain, and such distress can lead to systemic complications [12, 13]. To minimize these unpleasant conditions and complications, intravenous sedation has been widely used. The target depth of sedation is consistent with the American Society of Anesthesiologists’ definition of moderate sedation, where sedated patients are capable of purposeful response to verbal or tactile stimulation [1]. Existing studies defined adequate sedation as a Modified Observer’s Assessment of Alertness/Sedation scale score of 3, which indicates that the patient “responds only after (his/her) name is called loudly or repeatedly.” [14, 15]. This is likely an adequate level of sedation for therapeutic procedures. Furthermore, cardiovascular function and spontaneous ventilation are typically maintained in patients at this level of sedation, and no airway intervention is required [1].

The ideal properties of sedatives for procedural sedation are ease of use, rapid onset of action, quick recovery, and minimal residual sedation [16]. Benzodiazepine sedatives, of which midazolam is considered the gold standard [17], have been used for procedural sedation [1, 4, 19].

Midazolam is a short-acting GABA_A_ receptor agonist with an onset of action of 3–5 min and a potent amnesic effect. Midazolam is the most frequently used benzodiazepine [4]. However, its long half-life (1.8–6.4 h) results in longer sedation and less predictable recovery from sedation [18], which may affect the patient’s response to certain procedures, thereby affecting the doctor’s judgment regarding the effect of the procedure.

Remimazolam has dose-dependent sedative action with an onset of sedation within 60 s of administration [19]. The results of clinical trials indicate that remimazolam is more useful than midazolam for short procedural sedation, such as in patients who undergo colonoscopy, and that remimazolam’s safety profile is comparable to that of midazolam [3]. Remimazolam is expected to be safe and effective for a wide range of patients undergoing intravenous sedation for medical procedures.

For the induction and maintenance of procedural sedation in adults in the USA and the EU, the dosage of remimazolam should be individualized and titrated to the desired clinical response [19]. In the USA, the recommended dose of remimazolam for the induction of procedural sedation is 5 mg via an intravenous push injection over 1 min. If required, supplemental intravenous doses of remimazolam of 2.5 mg over 15 s may be given with ≥ 2 min between doses [2]. Opioids, such as fentanyl, are used as analgesics for successful sedation. In the EU, the recommended remimazolam dose regimen in adults receiving concomitant opioids (e.g., fentanyl 50 µg or sufentanil 5 µg) is consistent with the recommended US dosage (i.e., remimazolam 5 mg for the induction of procedural sedation and remimazolam 2.5 mg as a maintenance dose) [19]. While opioid use is more likely to induce respiratory depression and hypotension, there are no clinical studies evaluating the efficacy and safety of different sedatives combined with sufentanil in procedural sedation.

In our study, we used a single induction dose of remimazolam 5 mg combined with sufentanil 5 µg for procedural sedation and analgesia. We compared midazolam and dexmedetomidine with remimazolam to explore the efficacy and safety of the three regimens for procedural sedation during ultrasound-guided nerve block administration in patients undergoing abdominal tumor surgery.

From the perspective of improving perioperative management and patient satisfaction, multipoint nerve blocks are invasive procedures that should be performed under awake assisted sedation and analgesia before surgery. During the block, the depth of sedation should be appropriate to reduce preoperative stress and maintain stable vital signs. It is also necessary to restore the patient’s level of consciousness as soon as possible after the procedure. Additionally, cooperating with the anesthesiologist to evaluate the effect of the block is conducive to judging and evaluating the effect of the nerve block and improving patients’ satisfaction and perioperative anesthesia management. The results of our study showed that compared with midazolam and dexmedetomidine, remimazolam 5 mg combined with sufentanil 5 µg can quickly achieve the desired depth of sedation. Our results also showed that remimazolam was associated with a rapid onset and recovery and stable hemodynamics without increasing the incidence of respiratory depression and bradycardia. We assumed the higher incidence of hypoxemia with remimazolam compared with midazolam and dexmedetomidine may be related to remimazolam enhancing the sufentanil opioid analgesia; however, the underlying mechanism must be studied further and clarified.

In this study, we identified an interesting phenomenon regarding injection pain. Although there was no significant difference in the level of injection pain between the groups, clinically, many patients reported pain at the injection site in the remimazolam group. Pain on injection is one of the largest drawbacks of some sedatives, such as propofol [4]. One potential advantage of remimazolam may include low pain. In some studies, the degree of pain on injection was similar with remimazolam and midazolam. Remimazolam is a short-acting GABA_A_ receptor agonist. Its molecular formula is C21H19BrN4O2, with an average mass of 439.305 Da [17]. The structure of remimazolam is analogous to that of midazolam but with the addition of an ester moiety. Remimazolam is water-soluble, and consequently, there is less pain at injection sites than with fat-soluble agents. Regarding the slightly higher incidence of injection pain in the remimazolam group compared with the midazolam and dexmedetomidine groups, there are no studies investigating the mechanism underlying injection pain between different sedatives. The mechanism of injection pain must be studied further and clarified.

There are limitations in this study. This was a single-center investigation, and the sample size was relatively small, which limited the statistical analysis of the three groups of patients. Additionally, the mechanism underlying the slightly higher incidence of injection pain with remimazolam compared with the incidences with midazolam and dexmedetomidine was not studied.

## Conclusion

Remimazolam can be used safely for procedural sedation during ultrasound-guided nerve block administration in patients undergoing abdominal tumor surgery. The sedative effect is better than that with midazolam and dexmedetomidine, and sedation can be achieved quickly without obvious hemodynamic fluctuations. Compared with midazolam and dexmedetomidine, remimazolam is associated with better HR stability; slightly higher incidences of hypoxemia and injection pain (no statistically significant differences); and higher incidence of hypoxemia, which may be related to enhanced sufentanil opioid analgesia. The mechanism of injection pain with remimazolam must be studied further and clarified.This study was a single center study, and multicentre studies are recommended to reach more relevant conclusions.

## Data Availability

The datasets generated and analysed during the current study are not publicly available due to institutional restrictions but are available from the corresponding author on reasonable request.

## Abbreviations

ChiCTR: Chinese Clinical Trial Registry
BMI: Body mass index
R: Remimazolam
M: Midazolam
D: Dexmedetomidine
MAP: Mean arterial pressure
HR: Heart rate
SpO2: Pulse oxygen saturation
GABA-A: y-aminobutyric acid A
CONSORT: CONsolidated Standards Of Reporting Trials
TAPB: Transversus abdominis plane block
RSB: Rectus sheath block
TAP: Transversus abdominis plane
