# Correction: Efficacy and safety of remimazolam for procedural sedation during ultrasound-guided transversus abdominis plane block and rectus sheath block in patients undergoing abdominal tumor surgery: a single-center randomized controlled trial

**DOI:** 10.1186/s12871-023-02008-0

**Published:** 2023-02-07

**Authors:** Yimin Xiao, Ran Wei, Lanren Chen, Yunfei Chen, Lingsuo Kong

**Affiliations:** Department of Anesthesiology, Anhui Provincial Cancer Hospital, Huanhu East road 107, Shushan District, Hefei, 230022 China

**Correction: BMC Anesthesiol 22, 381 (2022)**

**https://doi.org/10.1186/s12871-022-01927-8**

Following publication of the original article [1], the authors noticed that they have made a mistake on the drug manufacture for remimazolam. Originally, the manufacturer of Remimazolam was indicated to have been Yichang Humanwell Pharmaceutical Co., Ltd by mistake. The authors noticed this, and the name of the manufacturer is now correctly states as Hengrui Pharmaceutical Co.,.

The original article [1] has been updated.
